# Evolutionary Relationships and Divergence of KNOTTED1-Like Family Genes Involved in Salt Tolerance and Development in Cotton (*Gossypium hirsutum* L.)

**DOI:** 10.3389/fpls.2021.774161

**Published:** 2021-12-14

**Authors:** Xiaohong Zhang, Junjie Zhao, Xiangyuan Wu, Genhai Hu, Shuli Fan, Qifeng Ma

**Affiliations:** ^1^Collaborative Innovation Center of Modern Biological Breeding, Henan Institute of Science and Technology, Xinxiang, China; ^2^State Key Laboratory of Cotton Biology, Institute of Cotton Research of CAAS, Anyang, China

**Keywords:** evolutionary, cotton, KNOX, stress response, artificial selection, development

## Abstract

The KNOX (KNOTTED1-like homeobox) transcription factors play an important role in leaf, shoot apical meristem and seed development and respond to biotic and abiotic stresses. In this study, we analyzed the diversity and evolutionary history of the *KNOX* gene family in the genome of tetraploid cotton (*Gossypium hirsutum*). Forty-four putative *KNOX* genes were identified. All *KNOX* genes from seven higher plant species were classified into KNOXI, KNOXII, and KNATM clades based on a phylogenetic analysis. Chromosomal localization and collinearity analysis suggested that whole-genome duplication and a polyploidization event contributed to the expansion of the cotton *KNOX* gene family. Analyses of expression profiles revealed that the *GhKNOX* genes likely responded to diverse stresses and were involved in cotton growth developmental processes. Silencing of *GhKNOX2* enhanced the salt tolerance of cotton seedlings, whereas silencing of *GhKNOX10* and *GhKNOX14* reduced seedling tolerance to salt stress. Silencing of *GhSTM3* influenced the cotton flowering time and plant development. These findings clarify the evolution of the cotton *KNOX* gene family and provide a foundation for future functional studies of KNOX proteins in cotton growth and development and response to abiotic stresses.

## Introduction

Cotton (*Gossypium* spp.) is the most important natural fiber source worldwide. *Gossypium hirsutum*, known as allotetraploid cotton, is among the most widely cultivated species and accounts for more than 90% of the global textile fiber production. The genome of *G*. *hirsutum* comprises the diploid A genome (*Gossypium arboreum*) and diploid D genome (*Gossypium raimondii*) derived from ancestral allopolyploidization (Zhang et al., 2015; Huang et al., 2020). The assembly of complete genome sequences for *Gossypium* species has provided substantial raw data, as well as a novel perspective of evolutionary conservation, divergence, and innovation in gene function in cotton.

Transcription factors are essential for the control of gene expression in plant developmental processes, such as leaf and floral development (McGarry et al., 2016; Zhang et al., 2016; Cheng et al., 2021), fiber elongation (Pei, 2015; Sun et al., 2019), biotic and abiotic stress responses (Shah et al., 2013; Yang et al., 2017), and hormone regulation (Li et al., 2014; Fiene et al., 2017). *KNOTTED1*-*like homeobox* (*KNOX*) genes belong to the three-amino-acid-loop-extension (TALE) superfamily and encode KNOX proteins with KNOXI, KNOXII, ELK, and homeobox KN binding domains (Bhatt et al., 2004). The first *KNOTTED1*-*like homeobox* gene to be identified, *Knotted 1* (*Kn1*), was isolated from a maize mutant (Smith et al., 1992). Additional *KNOX* homolog genes were identified from Arabidopsis and other plant species (Hareven et al., 1996; Long et al., 1996; Nadakuduti et al., 2014; Di Giacomo et al., 2017). On the basis of the similarities in the homodomain structure, Arabidopsis *KNOX* family genes can be divided into KNOXI, KNOXII, and KNATM clades. In Arabidopsis, KNOXI clade genes comprise *SHOOT MERISTEMLESS* (*STM*), *KNAT1*, *KNAT2*, and *KNAT6*. *STM* plays an important role in maintenance of apical meristem differentiation and floral development (Takano et al., 2010; Spinelli et al., 2011), broadened plant cell organwide growth and increased leaf complexity (Kierzkowski et al., 2019), and also regulates multiple floral fate genes (Roth et al., 2018). Arabidopsis fiber development is promoted by the plant hormone gibberellin and *KNAT1*, which is repressed by DELLA proteins (Felipo-Benavent et al., 2018). *KNAT2* plays an important role in carpel development (Pautot et al., 2001). The *knat6* mutation enhances the phenotype of the *stm-2* mutant, and reveals that *KNAT6* is involved in SAM maintenance and boundary establishment and modulates lateral root formation through the STM/CUC pathway (Dean et al., 2004; Belles-Boix et al., 2006). Expression of *KNAT2* and *KNAT6* may be restricted by the BP and PNY proteins to promote correct inflorescence development (Ragni et al., 2008). KNOXII clade genes of Arabidopsis comprise *KNAT3*, *KNAT4*, *KNAT5*, and *KNAT7*, which show diverse expression patterns in different organs, including roots, leaves, stems, and flowers (Truernit et al., 2006). *KNAT3* regulates seed germination and seedling growth through the abscisic acid signaling pathway (Kim et al., 2013a). KNAT7 interacts with OVATE FAMILY PROTEINS to influence secondary cell wall formation (Li et al., 2011, 2012), and orthologs of *KNAT7* expressed to varying degrees in fibrous wood species may explain differences in wood development (Reyes-Rivera et al., 2017). In cotton, the *KNAT7* homolog *GhKNL1* may partially rescue the phenotype of the Arabidopsis *knat7* mutant. *GhKNL1* encodes a protein that interacts with GhBEL1-like proteins to form heterodimers to regulate fiber development (Gong et al., 2014; Ma et al., 2019). Arabidopsis KNATM, which lacks the homeobox KN domain, is expressed in proximal-lateral domains of organ primordia and at the boundary of mature organs, and functions in leaf proximal-distal patterning (Magnani and Hake, 2008).

Additional research has revealed that *KNOX* genes are involved in abiotic stress responses. In soybean, most KNOXII genes exhibit higher expression levels in response to saline stress and dehydration (Wang et al., 2021). *GmSBH1*, a member of the KNOXI clade, is involved in the response to high temperature and humidity stress in soybean seed development (Shu et al., 2015). In *Populus*, the *KNOX* homolog gene *PagKNAT2*/*6b* alters plant architecture and improves drought resistance (Song et al., 2021). Wheat *LRD*, a *KNAT3* homolog gene, affects lateral root growth and grain size under water limitation (Placido et al., 2020). Although *KNOX* genes have been partly elucidated in plant development, and *KNOX* genes have been previously studied in cotton (Gong et al., 2014; He et al., 2021), our understanding of *KNOX* family members in cotton remains limited. In this study, we conducted a genome-wide analysis to identify 44 putative *KNOX* family members in *G. hirsutum*. In addition, gene expression patterns in specific tissues and in response to stress treatment were analyzed. A virus-induced gene silencing (VIGS) assay was used to study the function of *GhKNOX* genes. These results provide a basis for future investigations of the roles of *KNOX* proteins in plant development of cotton.

## Materials and Methods

### Sequence Identification

The complete *G. hirsutum* genome sequence data were obtained from COTTONGEN^1^ (Yu et al., 2014). The protein sequences of five additional plant species comprising *Physcomitrella patens*, *Selaginella moellendorffii*, *Oryza sativa*, *Theobroma cacao*, and *Populus trichocarpa* were retrieved from the JGI Phytozome database^2^ and Genbank database^3^. The amino acid sequences of KNOX proteins from *Arabidopsis thaliana*, which were used as query sequences to search for cotton KNOX ortholog proteins in local BLAST with BlastP (with an threshold value of *E* ≤ 1e-5), were accessed from TAIR 10^4^. Then, the collected KNOX-like candidate proteins were subjected to SMART for further selection based on their conserved domain^5^.

### Conserved Sequence and Phylogenetic Analysis

Multiple sequence alignment was performed with ClustalW^6^. The conserved KNOXI, KNOXII, and ELK domain sequences of cotton and Arabidopsis KNOX proteins were aligned. A phylogenetic tree was constructed from full-length KNOX amino acid sequences of seven plant species, using the neighbor-joining method combined with a bootstrap analysis and the Jones–Taylor–Thornton substitution model as implemented in MEGA7.0. Branch support was estimated by performing a bootstrap analysis with 1000 replicates (Tamura et al., 2007).

### Chromosome Location and Gene Structural Features

Chromosome size and gene location information for *GhKNOX* genes were extracted from the gene annotations (gff3) file accessible from the *Gossypium hirsutum* genome. MapChart 2.2 software was used to determine the distribution of the genes on the *G*. *hirsutum* chromosomes. The exon and intron structure was displayed using the GSDS 2.0 online server^7^. The collinearity of gene pairs in the *GhKNOX* family were mapped to generate a collinearity map using Circos software.

### Plant Growth and Stress Treatment

All upland cotton plants were grown in the field at the Henan Institute of Science and Technology. Different tissues were sampled from plants of the cultivar ‘TM-1.’ For stress treatments, seeds were sown in plastic pots under a 14 h light/10 h dark photoperiod at 28°C until the seedlings attained the second leaf expanded stage and were then treated with 20% polyethylene glycol 6000 (PEG) or 200 mM NaCl. Leaves were harvested at 0, 1, 3, 6, 12, and 24 h, immediately frozen in liquid nitrogen, and stored at –80°C for total RNA extraction. Shoot meristems were harvested from plants of the early maturing cultivar ‘Zao1’ and the late-maturing cultivar ‘CCRI50’ for RNA-sequencing (RNA-seq) from the fourth leaf expanded to the seventh leaf expanded stages. The raw reads were processed to retain only clean reads by removing the adaptor sequences, low-quality sequence reads (*Q* < 20), and poly-N stretches (>10%). The clean reads were mapped to the upland cotton reference genome to obtain unigenes using the Tophat2 software (Kim et al., 2013b). Expression of *KNOX* genes in different tissues and the cold, heat, salt and drought stress treatments was analyzed using raw RNA-seq data. The raw RNA-seq data were downloaded from the NCBI Sequence Read Archive^8^. The RNA-seq expression analysis was conducted using TopHat and Cufflinks. Gene expression was expressed as fragments per kilobase of transcripts per million mapped reads (FPKM). A heatmap was generated using TBtools (Chen et al., 2020).

### RNA Isolation and Quantitative Real-Time PCR

Total RNA was isolated from samples using a plant RNA purification kit (Tiangen). The first-strand cDNA was synthesized using the PrimeScript™ 1st Strand cDNA Synthesis Kit for RT-PCR (TaKaRa). Transcript levels were determined by quantitative real-time PCR (qRT-PCR) analysis using the Q6 Real-Time PCR System (Applied Biosystems) and SYBR Premix Ex Taq (2×) (TaKaRa). To normalize these samples, *GhACTIN* was as an endogenous control. Determination of reaction specificities and data processing were performed as described in previous study (Schmittgen and Livak, 2008). Gene-specific primers used for the PCR are listed in Supplementary Table 1. Three biological replicates were analyzed. The significance of differences between means was determined using analysis of variance implemented in SAS software (^∗^*P* < 0.05, ^∗∗^*P* < 0.01). The data were graphed using GraphPad Prism 5.

### Analysis of Genetic Variation and Artificial Selection of *Gossypium hirsutum KNOX* Genes

The basic information for 82 early and 67 modern cultivars from a core collection of upland cotton and the relative genomic variants were downloaded from the Hebei Agricultural University website^9^. Single-nucleotide polymorphisms (SNPs) in the *KNOX* genes were detected, based on the genomic location of the genes, and the number of SNPs per gene was scored using Excel 2010. The fixation statistic (*F*_st_) was calculated with Genepop 4.0 software (Rousset, 2008). The genes *F*_st_ > 0.45 were identified as putative sites under selection during improvement (Li et al., 2018).

### Virus-Induced Gene Silencing Assay and Stress Treatment

Based on previously described VIGS assay method (Gao et al., 2011), the genes *GhKNOX2-A*, *GhKNOX10-A*, *GhKNOX14-A*, *GhSTM2-A*, and *GhSTM3-A*/*D* were amplified from the ‘CCRI50’ cDNA library and inserted in the pCLCrVA vector. Gene-specific primers used for the VIGS assay are listed in Supplementary Table 1. The recombinant vectors were transformed separately into *Agrobacterium* strain GV3101. The GV3101 cells harboring recombinant plasmid were mixed with cells carrying pCLCrVB (1:1 ratio). The GV3101 cells were cultured then were injected into 10-day-old cotton cotyledons. The cotton plants were analyzed with regard to their gene expression profiles and phenotypes under salt stress. The inoculated cotton plants were grown in a greenhouse at 22°C under a 16 h light/8 h dark photoperiod. The content of malondialdehyde (MDA) and activity of peroxidase (POD) were assessed using a MDA assay kit and POD Assay Kit (Nanjing Jiancheng). The analysis was repeated three times, and each data type was analyzed from a sample of at least five plants in each independent biological experiment. The significance of differences between means was determined using Student’s *t*-test (^∗^*P* < 0.05, ^∗∗^*P* < 0.01).

## Results

### Identification of *KNOX* Genes in *Gossypium hirsutum*

We identified putative *KNOX* genes in the reference genome of *G. hirsutum*. Forty-four *GhKNOX* genes were identified. The *GhKNOX* genes were named on the basis of the similarity of the encoded amino acid sequence with that of Arabidopsis orthologs; ‘A’ and ‘D’ indicated derivation in the A and D subgenomes, and ‘a’ and ‘b’ were used to distinguish the corresponding paralogs of the same Arabidopsis ortholog. Thus, the 44 putative *KNOX* family genes were named *GhKNOX1* to *GhKNOX7* and *GhSTM1* to *GhSTM3*, and *GhKNL1* was identified in a previous study (Gong et al., 2014). The other genes identified had no highly orthologous counterparts in Arabidopsis and were named *GhKNOX8* to *GhKNOX14*. The cotton *KNOX* family genes encoded a peptide ranging in length from 102 to 865 amino acids, the molecular weight ranged between 11.31 and 98.44 KDa, and the isoelectric point value ranged from 4.08 to 8.81 (Table 1).

**TABLE 1:** Genomic information for *Gossypium hirsutum KNOX* family genes.

Gene name	Gene ID	Chromosome and location	Length (a.a.)	MW (Da)	Pi
GhKNL1-A	Gh_A08G1599	A08 94584210-94588505(+)	299	33.7	6.19
GhKNL1-D	Gh_D08G1910	D08 57040374-57044593(+)	300	33.8	6.23
GhKNOX1-A	Gh_A12G2447	A12 86968479-86970008(+)	161	18.5	6.4
GhKNOX1-D	Gh_D12G2573	D12 58614474-58616149(+)	161	18.5	6.82
GhKNOX2-A	Gh_A03G1199	A03 85855130-85862642(–)	314	35.4	5.06
GhKNOX2-D	Gh_D02G1633	D02 56604555-56612049(–)	314	35.4	5.06
GhKNOX3a-A	Gh_A06G0906	A06 35495471-35499464(–)	440	48.9	5.95
GhKNOX3a-D	Gh_D06G1066	D06 23144737-23148581(–)	433	48.2	5.98
GhKNOX3b-A	Gh_A05G0463	A05 5053139-5055793(–)	426	47.1	5.94
GhKNOX3b-D	Gh_D05G3920	scaffold4079_D05 28293-30932(+)	426	47.0	5.87
GhKNOX4a-A	Gh_A13G1595	A13 74847988-74853043(+)	468	51.5	6.41
GhKNOX4a-D	Gh_D13G1956	D13 54959586-54961686(+)	446	49.4	6.01
GhKNOX4b-A	Gh_A07G0245	A07 2973007-2974847(–)	434	48.0	5.87
GhKNOX4b-D	Gh_D07G0302	D07 3123345-3125202(–)	436	48.0	5.86
GhKNOX5a-A	Gh_A06G0419	A06 7209361-7210605(+)	214	23.6	5.23
GhKNOX5a-D	Gh_D06G0457	D06 6612100-6613730(+)	290	32.8	6.1
GhKNOX5b-A	Gh_A05G0046	A05 695203-697870(+)	295	33.3	5.35
GhKNOX5b-D	Gh_D05G0099	D05 1138017-1140642(+)	295	33.3	5.35
GhKNOX6-A	Gh_A05G2722	A05 48418454-48425193(–)	313	35.5	4.75
GhKNOX6-D	Gh_D05G3025	D05 39269297-39270458(–)	190	21.2	4.24
GhKNOX7a-A	Gh_A12G2049	A12 83413164-83415009(+)	303	34.7	6.36
GhKNOX7a-D	Gh_D12G2227	D12 55442059-55443895(+)	303	34.6	6.5
GhKNOX7b-A	Gh_A03G2005	scaffold503_A03 145324-148791(+)	299	33.6	5.8
GhKNOX7b-D	Gh_D03G1492	D03 44017402-44025542(–)	299	33.6	6.02
GhKNOX8-A	Gh_A05G1857	A05 19448909-19453532(–)	369	42.5	5.8
GhKNOX8-D	Gh_D05G2055	D05 18989805-18994433(–)	369	42.5	5.8
GhKNOX9-A	Gh_A06G1864	scaffold1256_A06 48478-78214(–)	865	98.4	8.81
GhKNOX9-D	Gh_D06G0225	D06 2240774-2244008(–)	364	41.8	6.14
GhKNOX10-A	Gh_A08G1820	A08 98869281-98872278(+)	233	26.0	5.21
GhKNOX10-D	Gh_D08G2181	D08 61364557-61367378(+)	290	33.2	5.66
GhKNOX11-A	Gh_A12G2495	A12 87356460-87362713(–)	303	34.2	5.55
GhKNOX11-D	Gh_D12G2623	D12 59002884-59009109(–)	303	34.1	5.73
GhKNOX12-A	Gh_A13G0926	A13 49193040-49196835(–)	313	35.5	5.17
GhKNOX12-D	Gh_D13G1173	D13 34976363-34980324(–)	313	35.4	5.31
GhKNOX13-A	Gh_A11G2492	A11 83623796-83628023(–)	320	36.2	4.75
GhKNOX13-D	Gh_D11G2813	D11 57851157-57855319(–)	320	36.3	4.73
GhKNOX14-A	Gh_A02G0822	A02 19252970-19255038(+)	310	35.7	7.7
GhKNOX14-D	Gh_D05G3026	D05 39271057-39271554(–)	102	11.3	4.08
GhSTM1-A	Gh_A05G1484	A05 15223234-15226453(–)	354	40.0	5.96
GhSTM1-D	Gh_D05G1655	D05 14836355-14839643(–)	353	39.8	6.1
GhSTM2-A	Gh_A06G1334	A06 94354787-94358301(–)	359	40.6	6.36
GhSTM2-D	Gh_D06G1663	D06 55238676-55241627(–)	357	40.3	6.36
GhSTM3-A	Gh_A10G0104	A10 849528-852319(+)	350	39.6	5.96
GhSTM3-D	Gh_D10G0108	D10 854619-857391(+)	353	40.1	6.09

### Phylogenetic and Structural Analysis of GhKNOX Proteins

**FIGURE 1:**
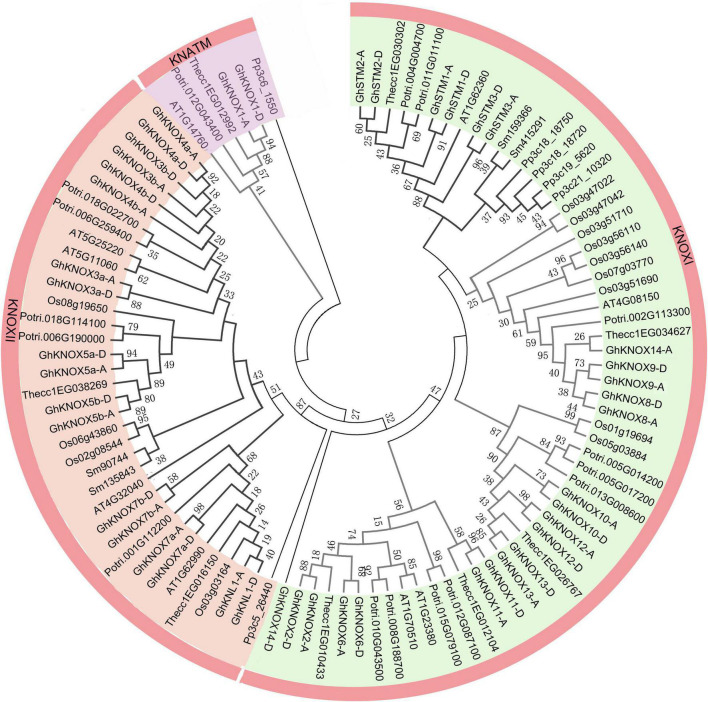
Phylogenetic relationship of KNOX family proteins of seven species. The phylogenetic tree was constructed from KNOX amino acid sequences using the neighbor-joining method with 1000 bootstrap replicates. The inner circle is marked in purple, orange, and green representing the KNOXI, KNOXII, and KNATM clades, respectively.

**FIGURE 2:**
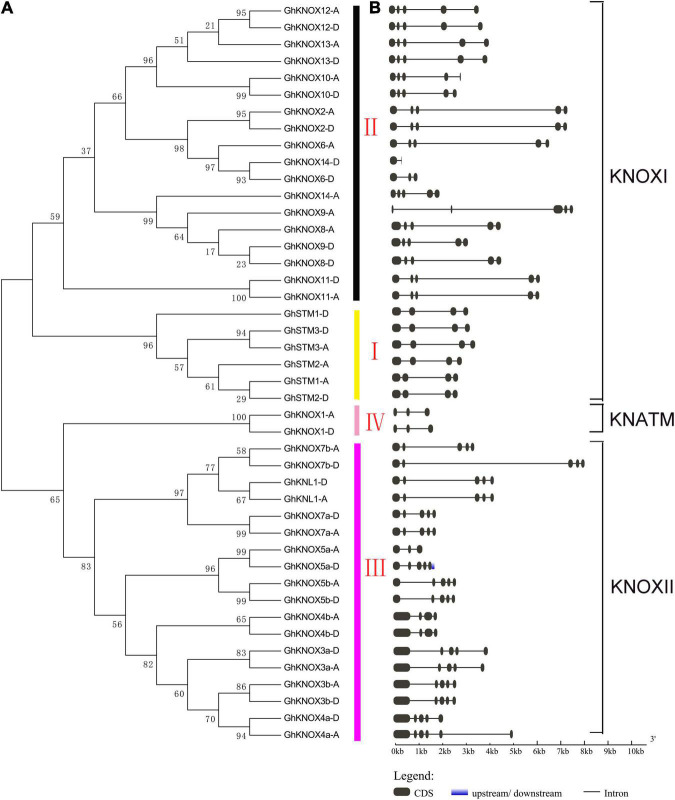
Phylogenetic relationships and genomic structure of *GhKNOX* genes. **(A)** Neighbor-joining tree of *GhKNOX*s. The *GhKNOX* genes were classified into four subclades (I, II, III, and IV). The subclades I and II were clustered in the KNOXI clade. The subclades III and IV belonged to the KNOXII and KNATM clades, respectively. **(B)** Exon–intron structural features of *GhKNOX* genes. Black boxes and lines indicate exons and introns, respectively.

To explore the evolutionary relationships of KNOX proteins among cotton and six other plant species, a neighbor-joining tree was constructed based on a multiple alignment of KNOX amino acid sequences. The KNOX proteins were divided into KNOXI, KNOXII, and KNATM clades (Figure 1). The KNOXI clade comprised the STM, KNAT1, KNAT2, and KNAT6 homologs derived from ferns, lycophytes, and angiosperms, and 24 GhKNOX proteins were clustered in this clade. The KNOXII clade comprised KNAT3, KNAT4, KNAT5, and KNAT7 homolog proteins. GhKNOX1-A/D were clustered in the KNATM clade. Most cotton KNOX proteins showed higher similarity with proteins from cacao and poplar; these genes were consistently clustered closely on one branch in the phylogenetic tree. Based on the classification of Arabidopsis KNOX proteins, subclades I and II belonged to the class KNOXI, and clades III and IV belonged to KNOXII and KNATM, respectively (Figure 2A).

Most (30 of 44) of the *G*. *hirsutum KNOX* genes contained four introns and five exons, and eight *KNOX* genes contained only three introns and four exons (Figure 2B). Only *GhKNOX14*-*D* incorporated one intron and two exons, and four genes (*GhKNOX1*-*A*/*D*, *GhKNOX5a*-*A*, and *GhKNOX6*-*D*) included two introns and three exons. The most highly similar exon and intron structures were observed in cotton genes within the same phylogenetic clade, thus supporting the reliability of the phylogenetic analysis. A multiple alignment of protein sequences was generated to detect the KNOX domain motifs in Arabidopsis and *G*. *hirsutum* (Supplementary Figures 1, 2). Four *G*. *hirsutum* proteins (GhKNOX1-A/D, GhKNOX5a-A, and GhKNOX6-D) contained only the KNOXI and KNOXII domains and lacked the ELK domain and homeobox KN binding domain. The GhKNOX10-A protein lacked the DNA-binding domain.

### Chromosomal Location and Synteny Analysis of *GhKNOX* Genes

**FIGURE 3:**
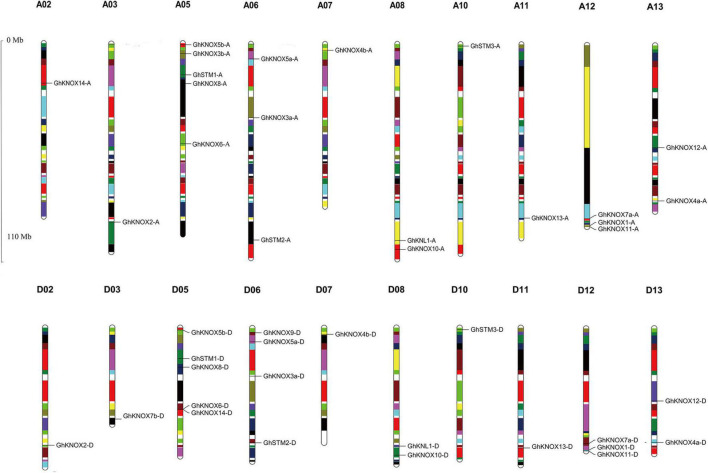
Physical locations of *KNOX* genes on *G. hirsutum* chromosomes. The upper and lower panels represent A subgenome chromosomes and D subgenome chromosomes, respectively. The scale is provided in megabases.

**FIGURE 4:**
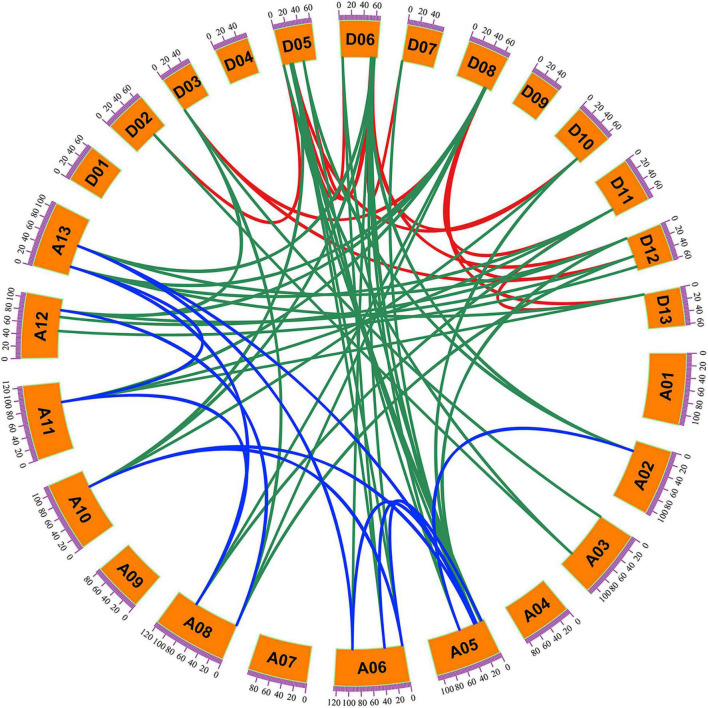
Collinearity analysis of *KNOX* genes on *G. hirsutum* chromosomes. Green lines link two homoeologous genes located in the A and D subgenome chromosomes. Red and blue lines link the two homologs formed by segmental duplication within the D subgenome and A subgenome, respectively. A01–A13 indicate the chromosomes in the A subgenome and D01–D13 indicate the chromosomes in the D subgenome.

Among the 44 *G*. *hirsutum KNOX* genes, 41 members were located on 20 of the 26 chromosomes assembled in the *G*. *hirsutum* genome v1.1, and the remaining three genes were located on three unmapped scaffolds (scaffold4079, scaffold503, and scaffold1256) (Figure 3). The number of *KNOX* genes per chromosome ranged from zero to five. Chromosomes A05 and D05 carried five genes, whereas no *KNOX* gene was detected on chromosomes A01/D01, A04/D04, and A09/D09. The *KNOX* genes located on homoeologous A and D chromosomes was conserved identical except for A02/D02, A03/D03, and A06/D06. The circos software was used to analyze *GhKNOX* gene duplication events in the upland cotton genome (Figure 4). The *GhKNOX* genes were unevenly distributed in A and D subgenomes, and specific duplications also occurred in the two subgeomes. More than ten *GhKNOX* genes were located in the A and D subgenome regions, respectively. Chromosomes A01/D01, A04/D04, A07, and A09/D09 did not contain any duplicated genes, whereas chromosomes A05/D05 and A06/D06 harbored the highest number of duplications. Chromosomes A03/D03 had three genes, but only one of them was paralog gene. Chromosomes D07 had one gene, while chromosomes A07 had no paralog gene. The collinearity analysis indicated that *GhKNOX* genes diverged from a common ancestor, but these genes were not conserved in the A and D subgenomes.

### Expression Pattern of *GhKNOX* Genes in Different Tissues and Shoot Meristem Developmental Stages

**FIGURE 5:**
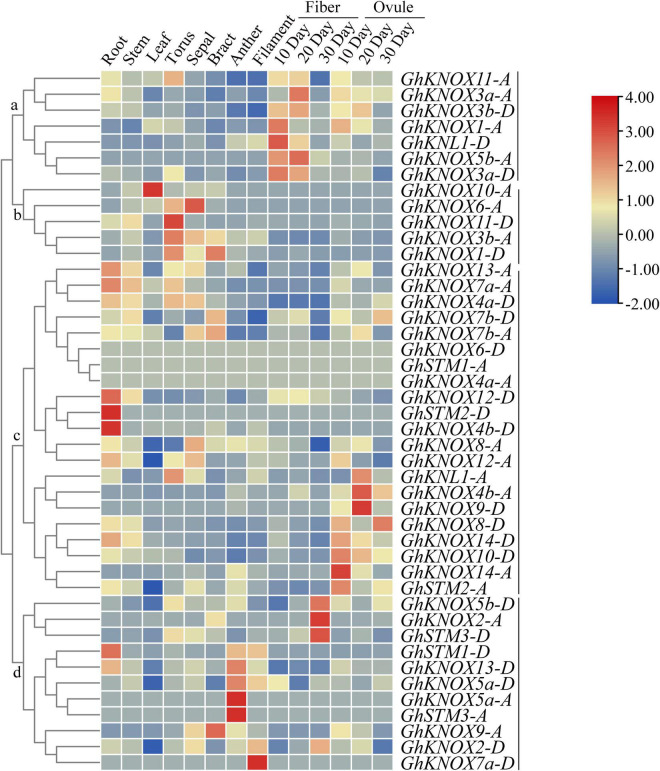
Expression patterns of *GhKNOX* genes in different tissues. The expression patterns were analyzed using hierarchical clustering. The FPKM values were calculated from RNA-seq data and are shown as a heatmap. The colored scale indicates the relative expression level.

Gene expression in different tissues may be associated with diversity in biological functions. The expression patterns of *GhKNOX* genes in ten organs (root, stem, leaf, torus, sepal, bract, anther, filament, fiber, and ovule) were analyzed (Figure 5). Among these genes, six genes (*GhKNOX1*-*A*, *GhKNL1*-*D*, *GhKNOX3a-A*/*D*, *GhKNOX3b-D*, and *GhKNOX5b*-*A*) in class a showed higher expression levels at different stages of fiber and ovule development. The class b genes *GhKNOX1*-*D*, *GhKNOX3b*-*A*, *GhKNOX6*-*A*, *GhKNOX10*-*A*, and *GhKNOX11*-*D* showed higher expression in floral organs, such as the torus, sepal, and bract. Most class c genes showed higher expression levels in tissues except fibers. Among these genes, *GhKNOX4b*-*D*, *GhKNOX12*-*D*, and *GhSTM2*-*D* were more highly expressed in the root, whereas *GhKNL1*-*A*, *GhKNOX4b*-*A*, *GhKNOX8*-*D*, *GhKNOX9*-*D*, *GhKNOX10*-*D*, *GhKNOX14*-*A*/*D*, and *GhSTM2*-*A* were predominantly expressed in ovules. The class d genes showed diverse expression patterns, which were focused on the root, sepal, anther, and filament. These results indicated that *GhKNOX* genes may have diverse biological functions in different tissues.

The expression patterns of *GhKNOX* genes in the shoot meristem of the early maturing cultivar ‘Zao1’ and the late-maturing cultivar ‘CCRI50’ were analyzed from the fourth leaf expanded to the seventh leaf expanded stages (Supplementary Figure 3). Eight genes in class A showed decreased expression levels at the four shoot apical development stages of ‘Zao1’ compared with those of ‘CCRI50.’ The transcript level of class B genes was highest at the fourth leaf expanded stage of ‘Zao1’ and at the seventh leaf expanded stage of ‘CCRI50.’ Other *GhKNOX* genes in class C exhibited higher expression levels in ‘Zao1’ with a lower expression level detected at the fourth leaf expanded stage. In ‘CCRI50,’ the majority of *GhKNOX* genes showed the highest transcript level at the sixth and seventh leaf expanded stages except *GhKNOX11-D*. Six *STM* homolog genes showed higher expression levels in ‘Zao1’ than in ‘CCRI50.’ Thus, the functions of *GhSTM* genes in cotton growth and development require further verification.

### Abiotic Stress Induced Expression Profiles of *GhKNOX* Genes

**FIGURE 6:**
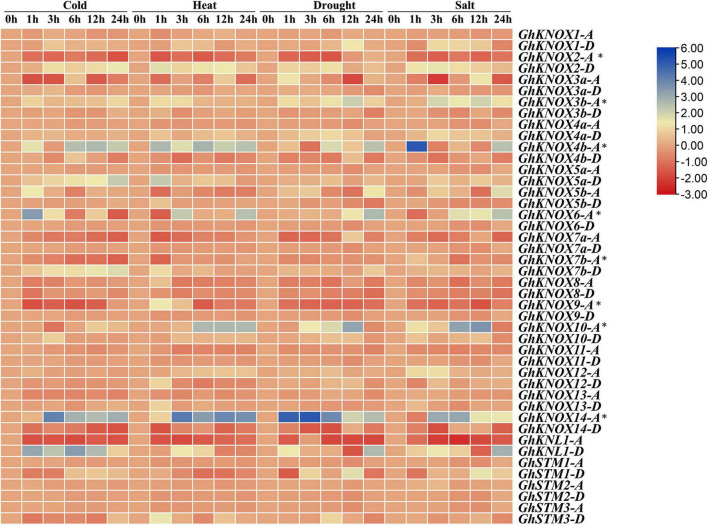
Expression profiles of *GhKNOX* genes in response to four abiotic stress treatments. The ratios of FPKM between the treatments (at 1, 3, 6, 12, and 24 h) and the control (at 0 h) were calculated from RNA-seq data and are shown as a heat map. The colored scale indicates the relative expression level. An asterisk indicates that expression of these genes requires verification by qRT-PCR.

The expression pattern of the 44 *GhKNOX* genes in response to exposure to cold, heat, salt, and drought stress was analyzed at different time points. The expression of some *KNOX* genes was affected significantly, such as *GhKNL1*-*D*, *GhKNOX2*-*D*, *GhKNOX3b*-*A*, *GhKNOX4b*-*A*, *GhKNOX6*-*A*, *GhKNOX10*-*A*, and *GhKNOX14*-*A*. The expression level of *GhKNOX2*-*D*, *GhKNOX4b*-*A*, *GhKNOX6*-*A*, and *GhKNOX14*-*A* was increased in response to the four stresses. The genes *GhKNOX2*-*A*, *GhKNOX14*-*D*, and *GhKNL1*-*A* showed decreased expression under the four stress treatments. Expression of *GhKNOX10*-*A* was not influenced by heat, drought, and salt stress. *GhKNOX5a*-*D* and *GhKNOX7b*-*D* showed higher expression levels in response to cold stress only, whereas expression of *GhKNOX5a*-*D*, *GhKNOX9*-*A*, and *GhSTM3*-*D* increased at 1 h and thereafter decreased slightly. The present results indicated that *GhKNOX* genes from the A subgenome displayed superior adaptability to environmental stresses (Figure 6).

**FIGURE 7:**
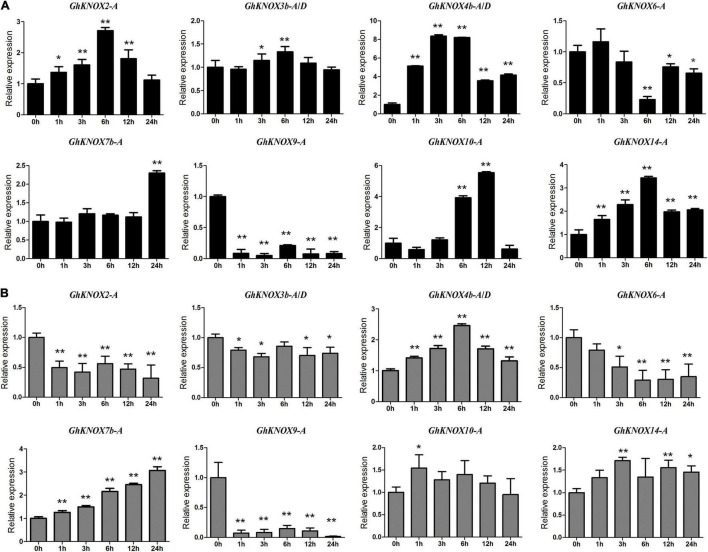
Expression patterns of selected *GhKNOX* genes in response to PEG or NaCl stress treatment. **(A)** Relative expression levels of *GhKNOX* genes between the control (0 h) and different time points (1, 3, 6, 12, and 24 h) under 20% PEG treatments. **(B)** Relative expression levels of *GhKNOX* genes between the control (0 h) and different time points (1, 3, 6, 12, and 24 h) under 200 mM NaCl treatment. *GhACTIN* (AY305733) was used as an internal control. Error bars indicate the standard deviation of three independent experiments. Relative expression was calculated using the 2^–△△*Ct*^ method. The significance of differences between means was determined using analysis of variance (*P < 0.05, **P < 0.01).

To explore the expression of *GhKNOX* genes in response to abiotic stresses in greater detail, we selected eight *GhKNOX* genes for which expression was induced by drought and salt stress, and examined their expression following treatment with 20% PEG or 200 mM NaCl. The qRT-PCR results showed that *GhKNOX4b-A*/*D*, *GhKNOX7b-A*, *GhKNOX10-A*, and *GhKNOX14-A* were upregulated by PEG or NaCl treatment. Transcription of *GhKNOX2-A* and *GhKNOX3b-A*/*D* was upregulated by PEG treatment and downregulated by NaCl treatment. *GhKNOX6-A*, and *GhKNOX9-A* were downregulated by PEG or NaCl treatment (Figure 7). These results implied that *GhKNOX* family genes may show differential expression levels under different abiotic stresses.

### Silencing of Five *KNOX* Homolog Genes in Cotton

**FIGURE 8:**
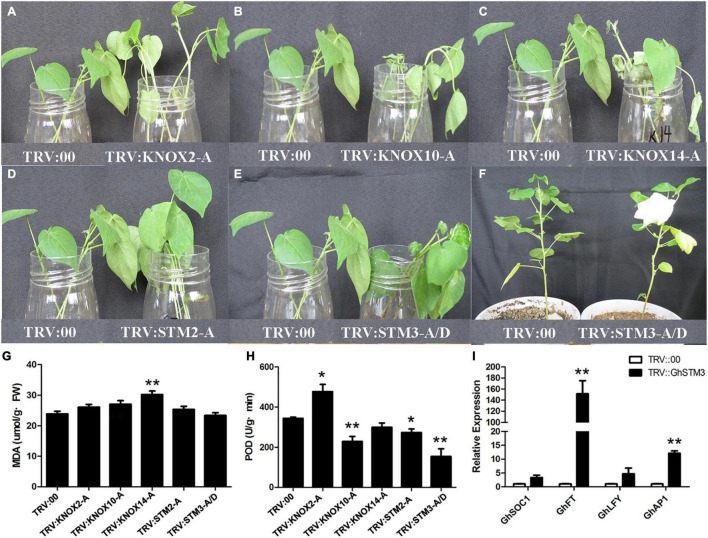
VIGS analysis of five *G*. *hirsutum KNOX* genes. **(A–E)** The left-hand plant is the control cotton transformed with the empty vector. In the right-hand plants, *GhKNOX2*-*A*, *GhKNOX10*-*A*, *GhKNOX14*-*A*, *GhSTM2*-*A*, and *GhSTM3*-*A*/*D* were silenced, respectively. **(F)** The control plant and *GhSTM3*-silenced plant of ‘CCRI50’ from left to right. **(G)** Malondialdehyde (MDA) content in TRV:00 and plants silenced for five *G*. *hirsutum KNOX* genes. **(H)** Peroxidase (POD) activity in TRV:00 and plants silenced for five *G*. *hirsutum KNOX* genes. **(I)** Relative expression levels of *GhSOC1*, *GhFT*, *GhLFY*, and *GhAP1*. The significance of differences was determined using Student’s *t*-test (*P < 0.05, **P < 0.01).

*GhKNOX2*-*A*, *GhKNOX10*-*A*, *GhKNOX14*-*A*, *GhSTM2*-*A*, and *GhSTM3*-*A*/*D* belonged to the clade KNOXI, which includes the Arabidopsis homolog genes *KNAT1*, *KNAT2*, *KNAT6*, and *STM*. The expression patterns implied that *GhKNOX2-A*, *GhKNOX10-A*, and *GhKNOX14-A* are induced by salt stress. We used VIGS assays to investigate the functions of these five *G. hirsutum* genes. The appearance of white leaves indicated that VIGS was successful and qRT-PCR analysis confirmed that the expression levels of the five *KNOX* genes decreased significantly in the VIGS plants (Supplementary Figure 4). Silencing of *GhKNOX2*-*A* increased salt tolerance, therefore the silenced cotton seedlings grew better than the control seedlings in response to salt treatment (Figure 8A). The POD activity of the silenced plants was significantly higher than that for control seedlings (Figures 8G,H). Silencing of *GhKNOX10*-*A* and *GhKNOX14*-*A* decreased the salt tolerance (Figures 8B,C), therefore the silenced cotton seedlings showed inferior growth compared with the control seedlings in response to salt treatment. The MDA content in *GhKNOX14-A* VIGS plants was significantly higher than that in control seedlings, whereas the POD activity of silenced *GhKNOX10-A* plants was lower than that of control seedlings (Figures 8G,H). Compared with control plants, VIGS of *GhSTM2-A* and *GhSTM3*-*A*/*D* did not result in significant changes in MDA content after salt treatment, whereas the POD activity decreased compared with that of the control (Figures 8D–H). The flowering time was promoted in *GhSTM3*-*A*/*D* VIGS plants, and expression of *GhFT* and *GhAP1* was upregulated with silencing of *GhSTM3*-*A*/*D* (Figures 8F,I). These results indicated that the five *KNOX* genes play an important role in salt stress tolerance and *GhSTM3* might affect the floral transition of cotton.

### Genetic Variations and Artificial Selection of *GhKNOX* Genes During Breeding Improvement

**FIGURE 9:**
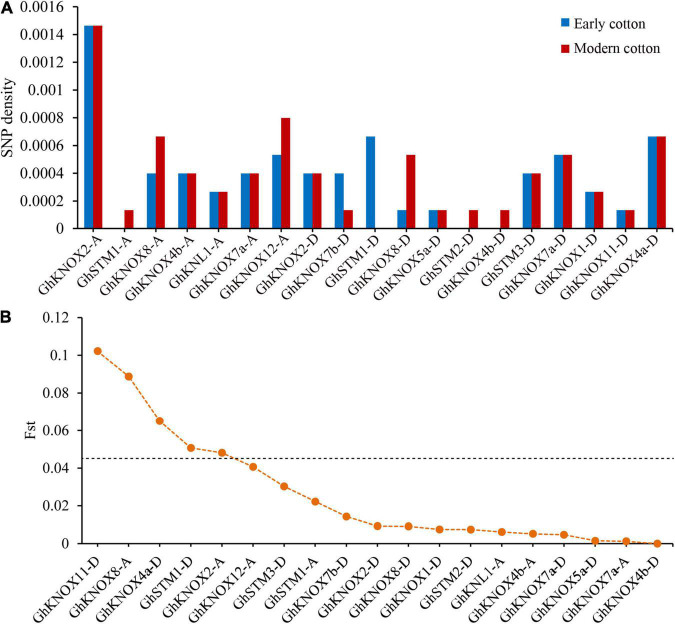
Genetic variation in *G*. *hirsutum KNOX* genes. **(A)** Density of SNPs in 19 *GhKNOX* genes. **(B)** The *F*_st_ values of the SNP loci in 19 *GhKNOX* genes. A *F*_st_ value for a SNP locus higher than 0.45 was considered to indicate putative sites under selection during domestication.

The increase in availability of resequencing data for cultivated cotton species enabled assessment of genetic differences in *KNOX* genes over several decades of breeding. In this study, we estimated the genetic variation of 82 early and 67 modern cultivars that were sequenced and the data released from a core collection of upland cotton (Ma et al., 2018). The early cultivars included introductions and cultivars bred before 1976, and the modern cultivars comprised those bred during the period 1996–2008. To compare genetic variation among different *KNOX* family genes in cotton cultivars, we counted the number of SNPs per gene. A total of 64 SNPs were detected in 19 *GhKNOX* genes and the number SNPs per gene ranged from 1 to 11. The early cultivars contained 54 SNPs in 16 *GhKNOX* genes, whereas modern cultivars contained 57 SNPs in 18 *GhKNOX* genes. The SNPs density of modern cultivars was higher than that of early cultivars for the genes *GhSTM1*-*A*, *GhSTM2*-*D*, *GhKNOX4b*-*D*, *GhKNOX8*-*A*, and *GhKNOX12*-*A*, whereas the reverse result was observed for *GhKNOX7b*-*D* and *GhSTM1*-*D*. These results showed that the *GhKNOX* genes exhibited rich genetic variation among both early and modern cultivars (Figure 9A).

To clarify the selective pressure exerted during breeding, we estimated the genetic difference among the two groups of cultivars (Figure 9B). There were distinct selective signals for *GhKNOX11*-*D* (0.10), *GhKNOX8*-*A* (0.089), *GhSTM1*-*D* (0.65), *GhKNOX2*-*A* (0.51), and *GhKNOX4a*-*D* (0.48) during cotton improvement, showing that these genes were subjected to intensive artificial selection. In contrast, 11 genes (*GhKNOX4b*-*A*, *GhKNL1*-*A*, *GhKNOX7a*-*A*, *GhKNOX2*-*D*, *GhKNOX7b*-*D*, *GhKNOX8*-*D*, *GhKNOX5a*-*D*, *GhSTM2*-*D*, *GhKNOX4b*-*D*, *GhKNOX7a*-*D*, and *GhKNOX1*-*D*) showed few genetic differences and an average *F*_st_ of 0.006. These results indicated that the latter genes have not been subjected to breeding selection and are potential improvement targets for breeders in the future.

## Discussion

*Gossypium hirsutum* is an allotetraploid species derived from hybridization between *G*. *arboretum* (A genome) and *G*. *raimondii* (D genome). A whole-genome duplication event occurred in the diploid species *G*. *raimondii* and *G*. *arboretum* (Huang et al., 2020). In the present study, we identified 44 *GhKNOX* genes in the cotton genome, which exceeds the nine *KNOX* genes identified in the Arabidopsis genome. Thus, the number of *KNOX* family genes has been expanded by approximately five-fold in cotton compared with that of Arabidopsis. The amino acid sequence alignment indicated that most GhKNOX proteins contained KNOXI, KNOXII, ELK, and homeobox KN binding domains except four proteins (GhKNOX1-A/D, GhKNOX5a-A, and GhKNOX6-D) that lacked the ELK and DNA binding domains, and GhKNOX10-A lacked the homeobox KN binding domain (Supplementary Figure 1). The ELK domain might be involved in transcriptional repression and function as a nuclear localization signal, and the homeobox KN binding domain located at the C-terminus is involved in DNA binding (Kerstetter et al., 1994; Nagasaki et al., 2001; Scofield and Murray, 2006). Thus, the five *GhKNOX* genes lacking these domains might have lost these respective functions.

Phylogenetic analysis revealed that *KNOX* genes were resolved into KNOXI, KNOXII, and KNATM clades. The KNOXI clade included the majority (58) of the *KNOX* genes, comprising two from *Selaginella moellendorffii*, four from *Physcomitrella patens*, four from Arabidopsis, ten from poplar, nine from rice, five from cacao, and 24 from cotton, whereas the KNOXII and KNATM clades consisted of 36 and six *KNOX* genes, respectively. Tandem and segmental duplications have been important for the expansion of gene families (Cannon et al., 2004). The expansion of gene number is important for adaptation to novel environments during plant evolution. *GhKNOX* family genes of cotton did not show tandem duplication, which is identical to Arabidopsis, *Populus*, and *Glycine* (Gao et al., 2015). Therefore, expansion of the *GhKNOX* gene family might have resulted from segmental duplication, and this reflects the adoption of novel functions in cotton. These differences suggest that the cotton *KNOX* gene family may have adapted to complex environmental conditions during evolution.

During plant evolution, *KNOX* genes have undergone major expansion from lycophytes to angiosperms, with not only increase in the large number of genes but also gene functional enrichment is apparent. The spatiotemporal expression patterns and functional analysis of *KNOX* genes have been studied in many species. The Arabidopsis *STM* gene is mainly expressed in the SAM and controls meristem formation and size (Aida et al., 1999; Spinelli et al., 2011). In the present study, *STM* homologs were strongly expressed in the SAM of the earlier-maturing cultivar ‘Zao1’ than that of the later-maturing ‘CCRI50.’ Also, *GhSTM* genes accumulated a number of SNP loci during evolution. Thus, we suggest that the function of these genes might be focused on plant growth and development. We used VIGS assays to investigate the functions of *GhSTM2*-*A* and *GhSTM3*-*A*/*D*. The results indicated that the flowering time was accelerated in *GhSTM3*-silenced cotton plants, and the expression levels of *FT* and *AP1* homologs were upregulated significantly. Previous research revealed that repression of *STM* by *AUXIN RESPONSE FACTOR* (*ARF*) genes in Arabidopsis may promote flower initiation, which is mediated by histone deacetylation (Chung et al., 2019). Our results indicated that *STM* might have a negative function in the regulation of flowering time, which might be regulated by ‘florigen’ and floral development-related genes. KNOXI clade genes in Arabidopsis regulate inflorescence architecture, leaf shape, and internode development (Douglas et al., 2002; Smith and Hake, 2003; Chang et al., 2019). *GhKNOX2-1* can interact with *ARF16* to regulate leaf shape during the diversification of cotton species (He et al., 2021). *PagKNAT2*/*6b*, a class I KNOX gene in *Populus*, could improve drought resistance by inhibiting the synthesis of gibberellin (Song et al., 2021). However, the role of *GhKNOX* genes in response to stress is still limited in cotton. In the present study, the KNOXI homolog genes *GhKNOX2*-*A*, *GhKNOX10*-*A*, and *GhKNOX14*-*A* were highly expressed in the fiber and ovule, and were regulated by salt and drought stress. The VIGS assay for *GhKNOX2-A* increased the activity of POD and salt tolerance, whereas silencing of *GhKNOX10-A* and *GhKNOX14-A* decreased salt tolerance by reducing the activity of POD and increasing the MDA content, respectively. Previous study concluded that the root of plant can not absorb water under saline environment, and partial genes participate in plant stress signals by osmotic adjustment, osmoprotection, and protein accumulation (Buchanan et al., 2015; Li et al., 2020). Therefore, whether *G. hirsutum* KNOXI genes perform diverse functions that affect abiotic stress response and plant growth and development requires further study.

The KNOXII clade genes *KNOX3*/*4*/*5*/*7* are involved in seed development and seed physical dormancy (Chai et al., 2016; Wang et al., 2020). The legume *KNOX3* gene regulates nodule formation through cytokinin biosynthesis and activation (Azarakhsh et al., 2015). The tomato *KNOX* gene *Tkn4* participates in pollen and pollen tube development and the regulation of plant growth through the gibberellin and auxin pathways (Yan et al., 2019). Rice *KNOX7* integrates secondary wall and cell growth master regulators in internode and panicle development (Wang et al., 2019). Most soybean KNOX II genes exhibited higher expression levels during saline stress (Wang et al., 2021). Consistent with these results, the present expression analysis of *GhKNOX3a*, *GhKNOX5b* and *GhKNL1* showed prior expression in the fiber, and *GhKNL1* affected fiber development in the secondary cell wall biosynthesis pathway (Gong et al., 2014). The KNOXII clade genes *GhKNOX4a*, *GhKNOX4b*, and *GhKNOX7b* were highly expressed in the root. We also observed that KNOXII clade genes, such as *GhKNOX3b*-*A*, *GhKNOX4b*-*A*, *GhKNOX5a*-*D*, *GhKNOX7b*-*D*, and *GhKNL1*-*D*, showed distinct responses to abiotic stresses. These results implied that *GhKNOX* genes might play an active role in stress response induction. Although expression patterns have been illustrated, the functional roles of these *KNOX* family genes remain to be clarified. Thus, the comprehensive analysis of *KNOX* genes described could supply valuable information to elucidate the gene family in cotton.

In this study, we used available bioinformatic data and methods to explore the evolutionary relationships and functional roles of cotton *KNOX* genes. Phylogenetic analysis demonstrated that *GhKNOX* genes were divided into three clades and were expanded during genetic evolution. Analyses of expression profiles and gene function indicated that the *GhKNOX* genes likely responded to diverse stresses and were involved in plant development of cotton. These results provide useful information for future functional studies of *KNOX* family genes in cotton.

## Data Availability Statement

The datasets presented in this study can be found in online repositories. The names of the repository/repositories and accession number(s) can be found in the article/Supplementary Material.

## Author Contributions

QM and SF conceived and designed research. XZ and JZ conducted the experiments and wrote the manuscript. GH and XW revised the manuscript. All the authors read and approved the manuscript.

## Conflict of Interest

The authors declare that the research was conducted in the absence of any commercial or financial relationships that could be construed as a potential conflict of interest.

## Publisher’s Note

All claims expressed in this article are solely those of the authors and do not necessarily represent those of their affiliated organizations, or those of the publisher, the editors and the reviewers. Any product that may be evaluated in this article, or claim that may be made by its manufacturer, is not guaranteed or endorsed by the publisher.

## Footnotes

1
http://www.cottongen.org

2
https://phytozome.jgi.doe.gov/pz/portal.html

3
https://www.ncbi.nlm.nih.gov/genbank/

4
http://www.arabidopsis.org

5
http://smart.embl-heidelberg.de/

6
http://www.ebi.ac.uk

7
http://gsds.gao-lab.org/

8
https://trace.ncbi.nlm.nih.gov/Traces/sra/

9
http://cotton.hebau.edu.cn/

## Supplementary Material

Supplementary Figure 1 Distribution of *G*. *hirsutum* KNOX protein motifs. The different-colored boxes represent the conserved motifs containing KNOXI (green), KNOXII (purple), ELK (cyan-blue), and homeobox KN (red) binding domains.

Supplementary Figure 2 Amino acid sequences showing the highly conserved KNOX domains in Arabidopsis **(A)** and *G*. *hirsutum* **(B)**.

Supplementary Figure 3 Expression profiles of *GhKNOX* genes at different stages of shoot meristem development in the *G. hirsutum* cultivars ‘Zao1’ and ‘CCRI50.’ Shoot apical buds were harvested at the fourth leaf expanded to the seventh leaf expanded stages. Colors from red to green represent expression levels ranging from high to low, respectively.

Supplementary Figure 4 Expression analysis of five *G*. *hirsutum KNOX* genes in the VIGS assay. **(A)** Phenotype of the positive control plant transformed with the pCLCrVA-PDS vector. **(B)** Relative expression levels in empty control (TRV:00) and five *GhKNOX* genes VIGS plants. The five *GhKNOX* genes were *GhKNOX2-A*, *GhKNOX10-A*, *GhKNOX14-A*, *GhSTM2-A*, and *GhSTM3-A/D*, respectively.

Supplementary Table 1 PCR primers used in the study.
