# Supplementary figures and images for: Evolutionary Relationships and Divergence of KNOTTED1-Like Family Genes Involved in Salt Tolerance and Development in Cotton (Gossypium hirsutum L.)
Source: Front Plant Sci. 2021 Dec 14;12:774161. doi: 10.3389/fpls.2021.774161 (PMC8712452; doi:10.3389/fpls.2021.774161)

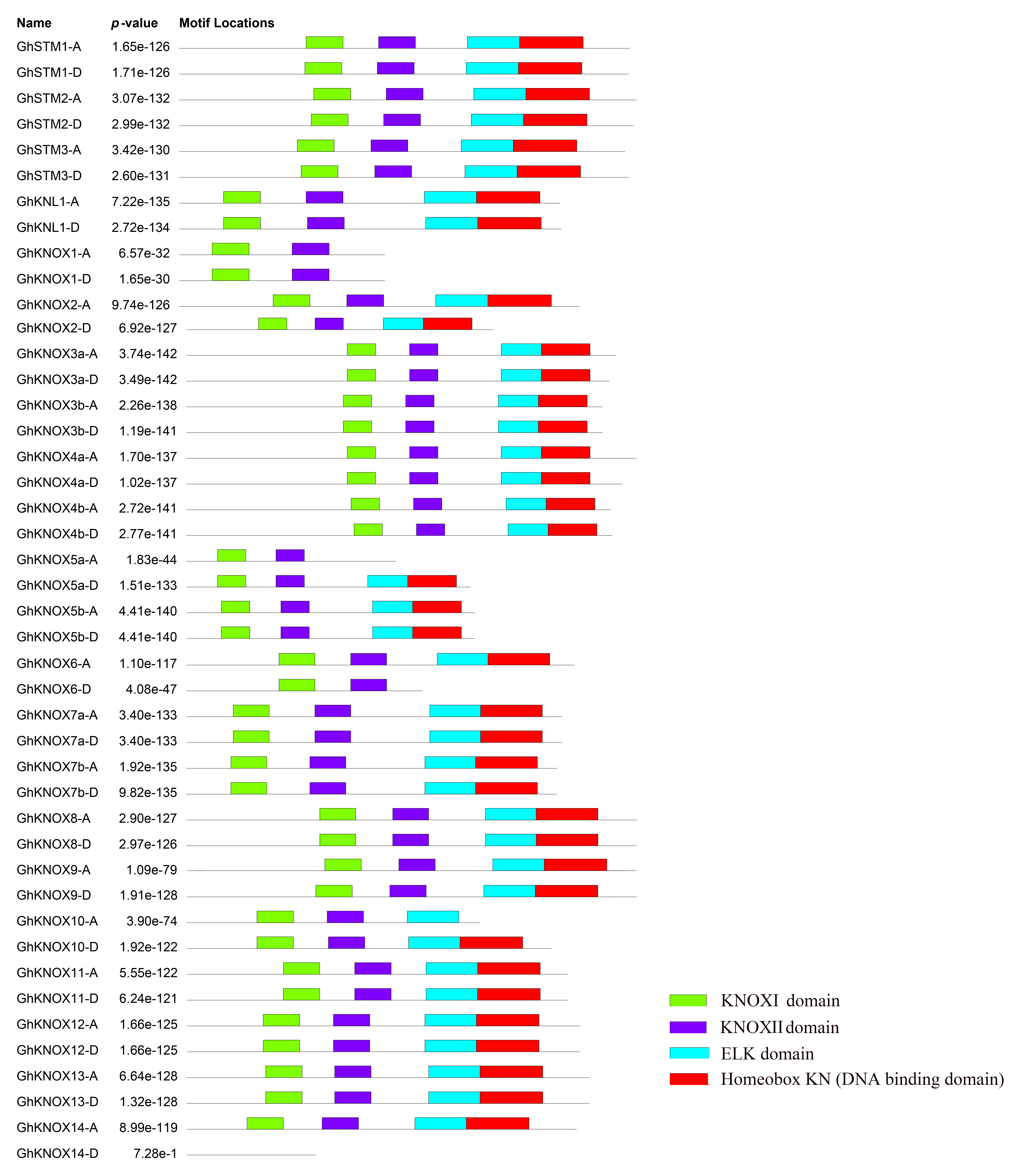

Supplement: Supplementary Figure 1 — Distribution of G. hirsutum KNOX protein motifs. The different-colored boxes represent the conserved motifs containing KNOXI (green), KNOXII (purple), ELK (cyan-blue), and homeobox KN (red) binding domains. [file Image_1.TIF]

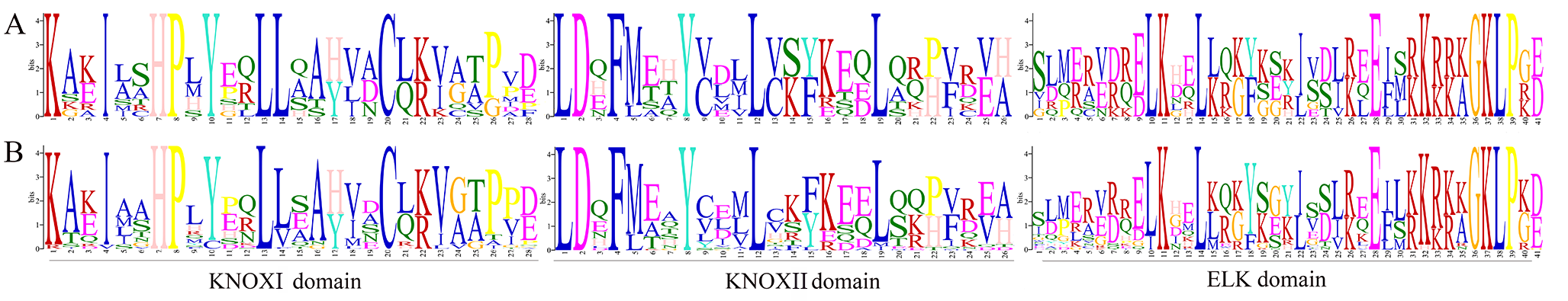

Supplement: Supplementary Figure 2 — Amino acid sequences showing the highly conserved KNOX domains in Arabidopsis (A) and G. hirsutum (B). [file Image_2.TIF]

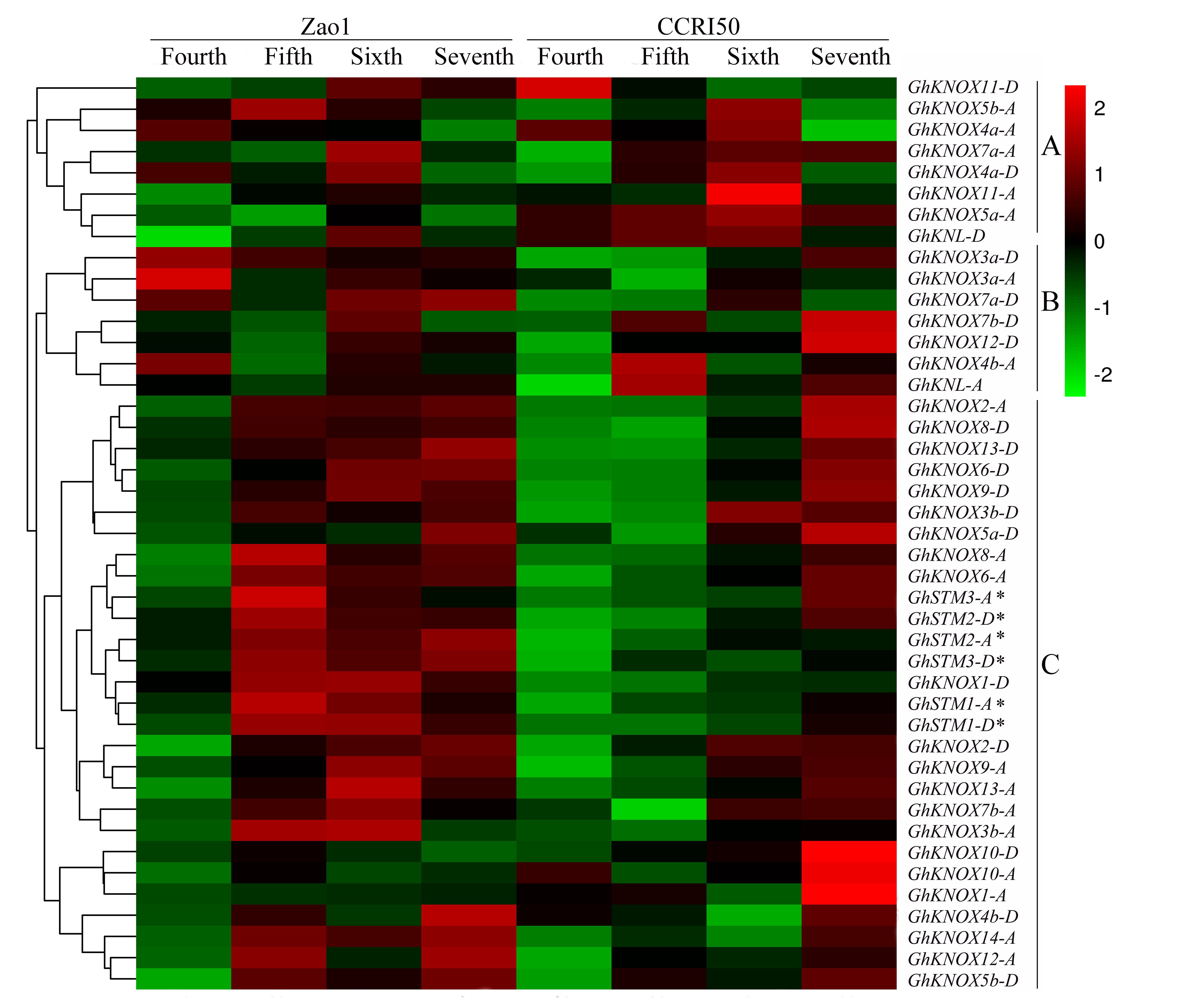

Supplement: Supplementary Figure 3 — Expression profiles of GhKNOX genes at different stages of shoot meristem development in the G. hirsutum cultivars ‘Zao1’ and ‘CCRI50.’ Shoot apical buds were harvested at the fourth leaf expanded to the seventh leaf expanded stages. Colors from red to green represent expression levels ranging from high to low, respectively. [file Image_3.TIF]

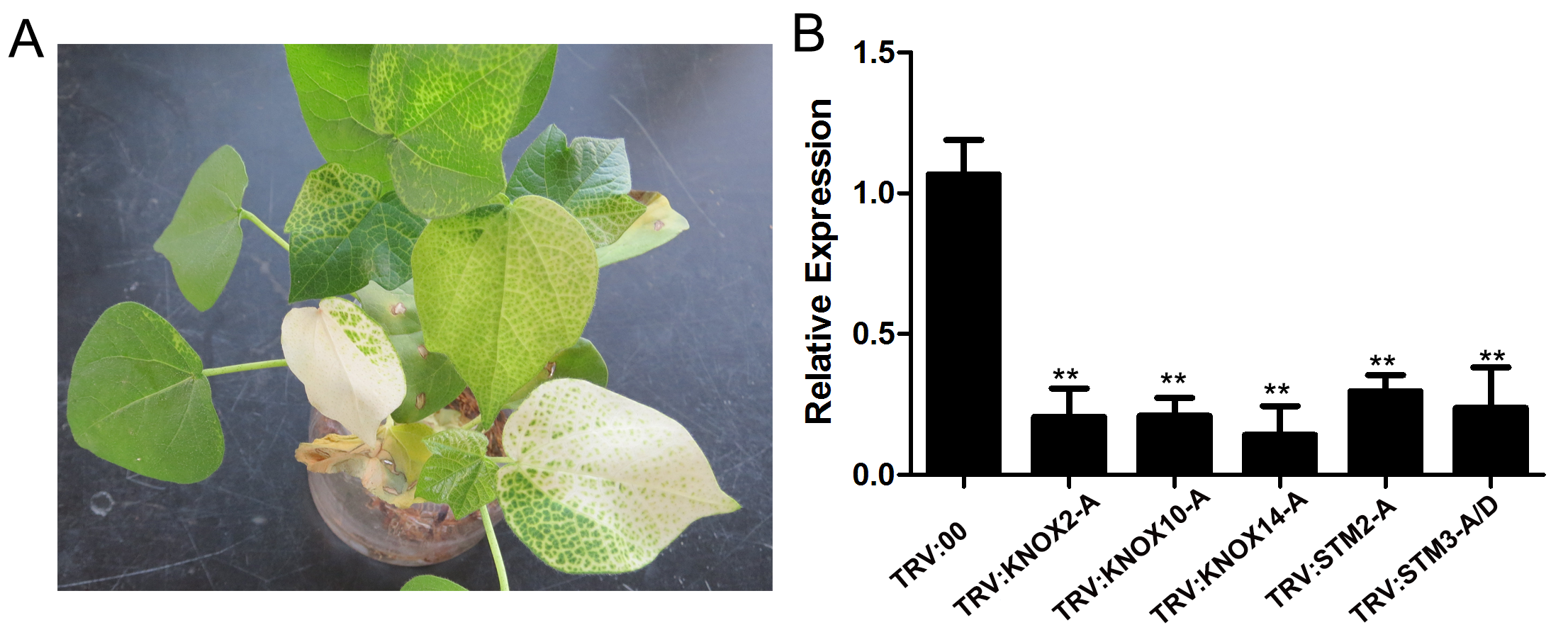

Supplement: Supplementary Figure 4 — Expression analysis of five G. hirsutum KNOX genes in the VIGS assay. (A) Phenotype of the positive control plant transformed with the pCLCrVA-PDS vector. (B) Relative expression levels in empty control (TRV:00) and five GhKNOX genes VIGS plants. The five GhKNOX genes were GhKNOX2-A, GhKNOX10-A, GhKNOX14-A, GhSTM2-A, and GhSTM3-A/D, respectively. [file Image_4.TIF]
